# Dental Laws and the State Board

**Published:** 1916-07

**Authors:** Martin Dewey


### DENTAL LAWS AND THE STATE BOARD.

BY MARTIN DEWEY, D.D.S., M.D.

As those engaged in the practice of dentistry in America increased in number, various laws in different states were passed to regulate the practice of that profession. The enactment of these laws was also influenced more or less by the advent of the dental college and the raising of the educational requirements by the National Association of Dental Faculties. The result of these efforts at regulation has been the establishment of state boards for the purpose of examining those who intend to practice dentistry. That the present methods of these boards are not satisfactory is proved by the many efforts that are being made to change the present laws and to place the state board examinations on a more uniform basis. Regardless of how efficient or how inadequate a law may be, there are always some persons to oppose a change. There is no question that the public should be concerned in the regulation of the practice of dentistry, as the laws are made for its protection, but practically all efforts for the enactment of medical or dental laws are promoted by the profession and not by the public. There has also been more or less disagreement between various organizations, and in some instances between state boards and dental colleges, which has caused needless conflicting laws in different states.

The medical profession has advocated the creation of a national examining board, which shall pass on the qualifications of every graduate in medicine, regardless of the state in which a graduate expects to locate. While this would probably be the ideal plan, a difficulty arises on account of the provisions of state laws, which give each state certain sovereign rights, especially in regard to police powers. If Congress would form a department—

known, we will say, as the Department of Public Health—which would be governed by a secretary the same as are the war, navy, and treasury departments, and medical and dental examinations be taken out of the hands of the states, and placed under the supervision of this department, matters would be greatly simplified. It is, however, needless to say there would be a great deal of opposition to such a plan, as it would be considered a centralization of power, would eliminate a large number of state boards, and cause the abolishment of offices occupied by certain men. It is rather unfair to expect a student who has been graduated from a medical or dental college to pass the state board examination in whatever state he expects to practice. If he is qualified to practice in one part of the United States, he should be qualified to practice in another. There seems to be no sound argument against this, and the only opposition comes from the resident members of the profession in a state. We have often heard it stated both by medical and dental men that they did not intend to have their locality overrun by men coming from other states to practice. It is known that in certain states of the United States it is practically impossible to pass the examination before the state board unless one is "a native son," or a graduate of a certain school located in the state in question. In other words, some state boards have assumed a protective power whereby they intend to protect the dental profession of the state from competition, regardless of the ability of the dentists in that state, or of the proficiency of the man who desires to locate there. This is a very unfair and narrow view but nevertheless that view prevails in some sections.

In regard to the status of orthodontia in respect to the state boards, we find a very undesirable condition existing. For example, a man may be qualified to practice orthodontia, having obtained that qualification either by years of practice or by a special line of training, and, if he desires to locate in another state, he is compelled to pass all the examinations required of a man who intends to practice general dentistry. It is desirable to have a certain examination for the man who is to specialize in orthodontia, and that examination should be of a character to determine his fitness for that specialty. It should be more exacting along certain lines than the examination required for the man who is to do general dentistry. It should also be compulsory for a man who intends to give up general practice and engage in orthodontia to present a recommendation or pass an examination to prove he is qualified to practice orthodontia as a specialty. Unfortunately, as conditions exist today, a man may be a failure in dentistry and decide over night that he will engage in orthodontia, and the public has no way of protecting itself. We have known of a few instances of men who qualified themselves to practice orthodontia, and decided to locate in states where they were not registered to practice dentistry, and, because they would compete with some man who had influence in a state and with the state board, they had considerable difficulty in obtaining a license. In one case a man was informed that if he would change the locality he had in view and go to another town, he would have no difficulty in passing the state board. These conditions point to the need of a more centralized plan of medical and dental examinations, and emphasize the necessity of having a special examination for those intending to engage in the practice of orthodontia, and of making every man pass that examination, whether he be native born or a foreigner. Just how this proposition is to be worked out will be a question of the future, and it is a question that demands a logical solution—*International Journal of Orthodontia.*

